# Presence of Mycotoxins in Milk Thistle (*Silybum marianum*) Food Supplements: A Review

**DOI:** 10.3390/toxins12120782

**Published:** 2020-12-08

**Authors:** Darina Pickova, Vladimir Ostry, Jakub Toman, Frantisek Malir

**Affiliations:** 1 Department of Biology, Faculty of Science, University of Hradec Kralove, Rokitanskeho 62, CZ-50003 Hradec Kralove, Czech Republic; ostry@chpr.szu.cz (V.O.); jakub.toman@uhk.cz (J.T.); frantisek.malir@uhk.cz (F.M.); 2 Center for Health, National Institute of Public Health in Prague, Nutrition and Food in Brno, Palackeho 3a, CZ-61242 Brno, Czech Republic

**Keywords:** milk thistle, food supplements, liver diseases, silymarin, mycotoxins

## Abstract

The consumption of herbal-based supplements, which are believed to have beneficial effects on human health with no side effects, has become popular around the world and this trend is still increasing. *Silybum marianum* (L.) Gaertn, commonly known as milk thistle (MT), is the most commonly studied herb associated with the treatment of liver diseases. The hepatoprotective effects of active substances in silymarin, with silybin being the main compound, have been demonstrated in many studies. However, MT can be affected by toxigenic micro-fungi and contaminated by mycotoxins with adverse effects. The beneficial effect of silymarin can thus be reduced or totally antagonized by mycotoxins. MT has proven to be affected by micro-fungi of the *Fusarium* and *Alternaria* genera, in particular, and their mycotoxins. Alternariol-methyl-ether (AME), alternariol (AOH), beauvericin (BEA), deoxynivalenol (DON), enniatin A (ENNA), enniatin A_1_ (ENNA_1_), enniatin B (ENNB), enniatin B_1_ (ENNB_1_), HT-2 toxin (HT-2), T-2 toxin (T-2), tentoxin (TEN), and zearalenone (ZEA) seem to be most significant in MT-based dietary supplements. This review focuses on summarizing cases of mycotoxins in MT to emphasize the need for strict monitoring and regulation, as mycotoxins in relation with MT-based dietary supplements are not covered by European Union legislation.

## 1. Introduction

According to the definition set by Directive 2002/45/EC, “Food supplements means foodstuffs the purpose of which is to supplement the normal diet and which are concentrated sources of nutrients or other substances with a nutritional or physiological effect, alone or in combination, marketed in dose form, namely forms such as capsules, pastilles, tablets, pills and other similar forms, sachets of powder, ampoules of liquids, drop dispensing bottles, and other similar forms of liquids and powders designed to be taken in measured small unit quantities.” [1]. The consumption of herbal-based food (dietary) supplements, which the manufacturers claim to have beneficial effects on human health, has become popular and has significantly increased over the last decade [2,3,4]. These herbal-based supplements are generally believed to be safer and healthier than synthetic drugs and free of side effects [4]. This may not always be the case, since herbal products can cause heavy liver damage leading to transplantation or even death [5]. One of the potential hazards lies in micro-fungi infestation of the plants, which can, as a result of inappropriate handling, storage and transport [6], lead to contamination with mycotoxins, which can persist in the final herbal supplementary products [4,7]. The presence of mycotoxins and other adulterants impairs the quality of supplements and thus the safety of their consumption [4,6]. The dishonesty of some manufacturers allows these reduced quality, and in the worst case potentially harmful, products to be marketed [4].

Milk thistle (MT) is a wild thorny herb considered a weed in many areas (see Section 2). Supplements based on this herb are among the top-selling herbal supplements in the US in the mainstream multioutlet channel. In 2018, it was the 20th best-selling herbal supplement with total sales of 16.6 million US dollars. However, compared to 2017, sales decreased by 1.6% [8]. MT is the most commonly researched herb associated with the treatment of liver disease [9], the cause of approximately two million deaths worldwide each year, accounting for 3.6% of all deaths worldwide [10]. However, its main biologically active compound, silymarin (see Section 3) has been proven to have many beneficial effects (see Section 4). Nevertheless, using modern analytical methods (see Section 5), infestation with various micro-fungi [3,11,12,13,14] and contamination with their mycotoxins [2,3,15,16,17] in MT-based supplements has been reported in several studies (see Section 6 and Section 7). The highest multi-mycotoxin concentration found in MT-based supplements has reached up to 37.6 mg/kg in total [3]. This concentration slightly exceeds the value earlier determined in the study by Veprikova et al. [15].

Mycotoxins are produced by various micro-fungi as their secondary metabolites, with no biochemical significance in microfungal growth and development [18]. Although they are harmless to their producers, they can elicit adverse effects (carcinogenic, genotoxic, hepatotoxic, teratogenic, estrogenic, immunosuppressive, nephrotoxic, or neurotoxic) in other organisms, mainly in humans and/or animals upon the consumption of contaminated food/feed [18,19]. Some of the mycotoxins produced by *Alternaria* or *Fusarium* species have been shown to be significant in MT-based supplements (see Section 8). Although the occurrence of mycotoxins in herbal-based food supplements is not negligible, they are not yet regulated in EU legislation (see Section 9). This situation needs to be further monitored. Exposure assessment is also needed, but studies on this topic are scarce (see Section 10).

In this review, a total of nine relevant original papers [2,3,11,12,13,14,15,16,17] concerning mycotoxins and/or micro-fungi have been included. All these publications were published in the period 2009–2019.

## 2. Botanical Description

*Silybum marianum* (L.) Gaertn. (syn. *Carduus marianus* L.) is commonly known as milk thistle but is known by many other names such as blessed milk thistle, Blessed virgin thistle, Christ’s crown, heal thistle, holy thistle, Marian thistle, Mary thistle, Saint Mary´s thistle, our lady´s thistle, sow thistle, variegated thistle, venue thistle, or wild artichoke [9,20]. It is a wild thorny annual or, rarely, biannual plant of the *Asteraceae* family [20,21,22], in many areas considered a weed due to its competitive and aggressive growth, usually reaching a height of 90-200 cm, but even up to 300 cm [23,24]. Purple flower heads and green leaves with milky white veins and strong spiny edges are typical features of the plant. The fruits are black achenes with oily eliosome that has significance in myrmecochory–dispersal by ants [24]. The plant originates in the Mediterranean basin, but it has spread to central Europe, America and South Australia [24] and nowadays is found worldwide [20,24]. 

## 3. Bioactive Compounds of Milk Thistle

**Figure 1:**
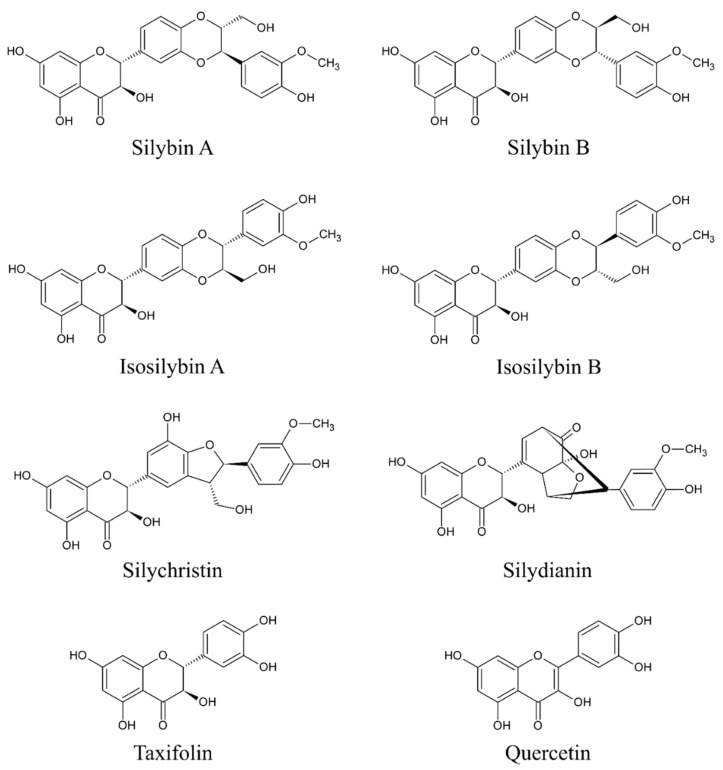
Chemical structures of main flavonolignans and flavonoids contained in silymarin complex.

The main bioactive complex of MT, collectively known as silymarin, consists mainly of flavonolignans (silybin A (PubChem Compound Identification Number /CID/: 31553), silybin B (PubChem CID: 1548994), isosilybin A (PubChem CID: 11059920), isosilybin B (PubChem CID: 10885340), silydianin (PubChem CID: 11982272), and silychristin (PubChem CID: 441764)), flavonoids (taxifolin (PubChem CID: 439533) and quercetin (PubChem CID: 5280343)), and polyphenolic compounds [25,26,27,28]. However, silybin (syn. silibinin) is considered the main bioactive component [23,25] as it accounts approximately for 50%–60% of silymarin [9]. The content of other components is approximately 5% for isosilibyn, 20% for silychristin, and 10% for silydianin and other compounds such as silimonin, isosilychristin, and isosilibinin [9]. Although silymarin is present throughout the whole plant, the highest concentration is found in the seeds [9,22]. The chemical structures of the eight above-mentioned flavonolignans and flavonoids are depicted in Figure 1.

## 4. Beneficial Effects of Milk Thistle-Based Supplements

MT has been used as a therapeutic herb for 2000 years [25]. Its main compound silymarin is without a doubt the most popular, most well-researched and potentially most effective herbal product used in the treatment of liver disease in particular, including toxin-induced liver disease, viral hepatitis, liver cirrhosis and hepatocellular carcinoma [5,9,15,25]. In addition, MT is also used in the treatment of kidney, spleen and biliary diseases [25,29]. Besides its well-known hepatoprotective properties, silymarin has also been shown to have antioxidant, antifibrotic, anti-inflammatory, choleretic, and immune-stimulating, regenerative, cytoprotective, cardioprotective, neuroprotective, anti-carcinogenic properties [9,25,29,30]. MT can be used as an antidote or a protective agent against both chemical (metals, fluoride, pesticides, cardiotoxins, neurotoxins, hepatotoxins, and nephrotoxins) and biological (snake and scorpion venoms, bacterial toxins, and mycotoxins) xenobiotics [30]. Due to this wide range of beneficial effects, many recent studies have focused on the effects of silymarin on various health problems. Several studies have demonstrated the neuroprotective effects of silymarin and its potential use in the treatment of Alzheimer’s disease [31,32]. Furthermore, positive effects of silymarin in the treatment of prostatic disorders such as benign prostatic hyperplasia [33], in decreasing frequency and severity of menopausal hot flashes [34], or in alleviating the side effects of the chemotherapeutic drug doxorubicin [35,36] have been demonstrated. The possible use of silymarin against solar-induced skin ageing has been demonstrated in a recent study [37]; however, Fidrus et al. warn of increased UVA-induced cytotoxicity after silymarin treatment [38]. Moreover, enhanced proteosynthesis, liver regeneration, increased lactation and immunomodulatory activity have also been associated with the effect of silymarin [9].

The efficacy of silymarin against the adverse effects of some mycotoxins has also been reported. As reviewed by Alhidari et al. (2017), many studies have demonstrated the beneficial effect of silymarin on aflatoxin B_1-_ (AFB_1_)-induced reduction of feed intake and weight gain of broilers [39]. Additionally, silymarin has completely prevented the ochratoxin A- (OTA)-induced immunosuppressive effect and has exerted hepatoprotective and nephroprotective effects in broiler chicks [40]. In a recent study, silymarin has been reported to provide cytoprotective activity against OTA, fumonisin B_1_ (FB_1_) and deoxynivalenol (DON) in porcine kidney-15 (PK-15) cells [41]. The alleviating effect of silymarin on zearalenone (ZEA)-induced liver damage and reproductive toxicity in rats has also been reported [42].

MT is marketed as a “dietary supplement” in various forms including seeds, capsules, tablets, granules, extracts or teas. Producers tend to specify the amount of the plant extract contained in the supplement. However, the content of active compounds in the extract itself can vary depending on the conditions (temperature, climate, season, soil, etc.) in which the plant was grown [4]. The recommended daily dose (RDD) of silymarin usually ranges from 420 mg to 600 mg, depending on the application defined by the manufacturer. The most common usage is in three doses of 140 mg of silymarin [43]. As demonstrated in a study by Fenclova et al., the content of silymarin compounds can vary considerably (5–393 mg/g), throughout various supplements as well as inter-batch [3]. The inconsistency of the number of bioactive compounds may lead to a reduced effect or to an overdose [4], which is manifested with gastrointestinal discomfort (nausea, diarrhea, abdominal pain, etc.) [29]. 

However, the biomass of MT can also be used in a non-medicinal way, including e.g., human and animal nutrition, bioenergy production, phytoremediation, agriculture, or cosmetic industry [21]. The supplementation of feed with MT/silymarin has proven useful in the livestock diet. The improved growth rate and meat quality in pigs [44] and rabbits [45] and increased milk yield and/or quality in cows [46] and sheep [47] have been linked to such supplementation. Moreover, an increase in the egg yield was observed in hens whose feed has been supplemented with MT [48].

## 5. Methods Used in the Determination of Mycotoxins in Milk Thistle-Based Dietary Supplements

The extraction of mycotoxins from the matrix of MT-based dietary supplements was usually based on the “quick easy cheap effective rugged safe” (QuEChERS) approach [2,3,15] or the dispersive liquid-liquid microextraction (DLLME) approach [2] followed by analysis performed by ultra-high-performance liquid chromatography coupled with tandem mass spectrometry (UHPLC-MS/MS) in studies by Arroyo-Manzanares et al. and Veprikova et al. [2,15], or high-resolution mass spectrometry (UHPLC-HRMS) in a study by Fenclova et al. [3]. A clean-up step based on the immunoaffinity columns followed by separation and quantification using reversed-phase liquid chromatography (RPLC) and determination by post-column photochemical derivatization and fluorescence detection (FLD) was employed in a study by Tournas et al. [17]. The enzyme-linked immunosorbent assay (ELISA) method was used after a clean-up step using multifunctional or polyamide columns in a study by Santos et al. [16]. For more details concerning methods used in the determination of mycotoxins in milk thistle-based dietary supplements see Table 1.

## 6. Micro-fungi in Milk Thistle-Based Dietary Supplements—An Overview

MT has been shown to be infested with numerous saprotrophic and potentially pathogenic molds. *Alternaria* genus, mainly *A. alternata*, is the most prevalent [11,12,13,14]. The occurrence of *Aspergillus* spp., *Eurotium* spp., *Melanospora* spp., *Mortierella* spp., *Mucor* spp., *Rhizopus* spp., *Ulocladium* spp., *Verticillium* spp., and *Zygorhynchus* spp. is also significant, while the occurrence of *Botrytis* spp., *Phoma* spp., and *Rhizoctonia* spp. is seen rather less often [11,13]. *Cladosporium* spp., *Fusarium* spp., and *Penicillium* spp. have also been found predominant in a study by Rosinska et al. [13], while less often in other studies [11,14]. Other fungi species from the genera of *Acremoniella* spp., *Acremonium* spp., *Arthrinium* spp., *Bipolaris* spp., *Chaetomium* spp., *Epicoccum* spp., *Monascus* spp., *Gliomastix* spp., *Humicola* spp., *Paecilomyces* spp., *Papulaspora* spp., *Phialophora* spp., *Phomopsis* spp., *Sordaria* spp., *Sporotrichum* spp., *Stagnospora* spp., *Stemphylium* spp., *Thamnidium* spp., *Trichoderma* spp., and *Trichothecium* spp. have also been isolated from MT [3,11,12,13,14].

The different maximum limits for molds in various herbal materials, based on their intended use, have been set at three levels [49]: the limit of 10^5^ colony forming units per gram (CFU/g) for “Raw medicinal plant and herbal materials intended for further processing”, 10^4^ CFU/g for ”Herbal materials that have been pretreated” and “Herbal medicines to which boiling water is added before use”, and 10^3^ CFU/g for “Other herbal materials for internal use” and “Other herbal medicines” [49]. In a study by Tournas et al. [14], *Aspergillus flavus, A. foetidus, A. penicillioides, A. versicolor, Eurotium amstelodami,* and *E. repens* have exceeded the limit of 10^5^ CFU/g. *Alternaria* spp., *Aspergillus candidus*, *A. niger*, *A. tritici*, *Eurotium* spp., *E. rubrum*, *Fusarium* spp., *Fusarium proliferatum*, and *Penicillium chrysogenum* have met or exceeded the limit of 10^4^ CFU/g. *Aspergillus* spp., *A. parasiticus*, *A. sydowii*, *A. tamarii*, *A. tubingensis*, *Penicillium* spp., *P. diercxii*, *Rhizopus* spp., *Fusarium subglutinans*, and *Eurotium chevalieri* have met or exceeded the limit of 10^3^ CFU/g.

## 7. Mycotoxin Contamination of Dietary Supplements Based on Milk Thistle—An Overview

This review provides a summary of five original papers on mycotoxins in various forms of dietary supplements based on MT. The results of the individual original papers have been summarized to create a comprehensive analysis. For the purpose of this review, the various forms have been grouped into six categories as follows: (1) seeds, (2) capsules, (3) tablets, (4) granules, (5) extracts, and (6) herbs. 

Throughout all five original studies, a total of 57 mycotoxins have been tested in various MT-based supplements, namely: 3-acetyl deoxynivalenol (3-AcDON), 3/15-acetyl deoxynivalenol (3/15-AcDON), aflatoxins (AFs), AFB_1_, aflatoxin B_2_ (AFB_2_), aflatoxin G_1_ (AFG_1_), aflatoxin G_2_ (AFG_2_), agroclavine (AGC), alternariol-methyl-ether (AME), alternariol (AOH), beauvericin (BEA), citrinin (CIT), cyclopiazonic acid (CPA), diacetoxyscirpenol (DAS), DON, deoxynivalenol-3-glucoside (DON-3G), enniatin A (ENNA), enniatin A_1_ (ENNA_1_), enniatin B (ENNB), enniatin B_1_ (ENNB_1_), ergot alcaloids (EA; including ergocornine, ergocorninine, ergocristine, ergocristinine, ergocryptine, ergocryptinine, ergometrine, ergosine, ergosinine, ergotamine, ergotaminine), fumonisins (FBs), FB_1_, fumonisin B_2_ (FB_2_), fumonisin B_3_ (FB_3_), fusarenon X (FUS-X), gliotoxin (GLI), HT-2 toxin (HT-2), meleagriin (MEL), mycophenolic acid (MPA), neosolaniol (NEO), nivalenol (NIV), OTA, patulin (PAT), paxilline (PAX), penicillic acid (PeA), penitrem A (PenA), phomopsin A (PHO-A), roquefortine C (ROC), sterigmatocystin (STEG), stachybotrylactam (STLAC), T-2 toxin (T-2), tenuazonic acid (TEA), tentoxin (TEN), verrucarol (VER), verruculogen (VERR), ZEA, α-zearalenol (α-ZOL), β-zearalenol (β-ZOL).

**Figure 2:**
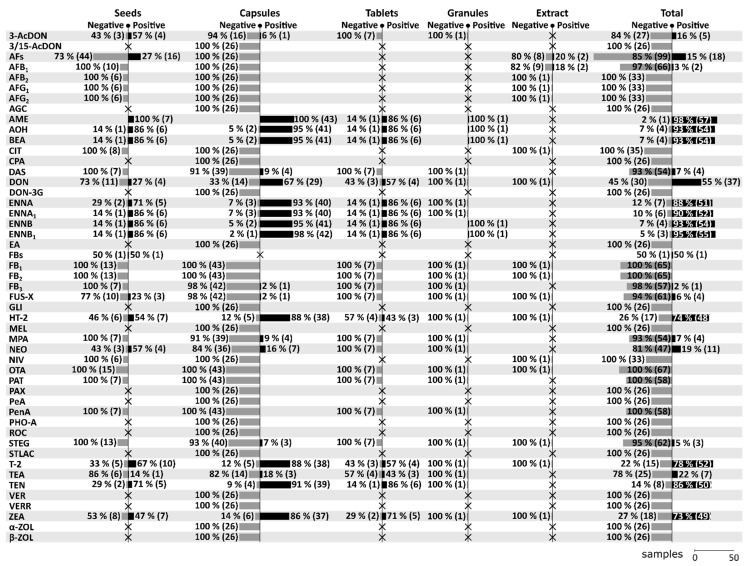
Contamination of milk thistle-based dietary supplement depending on its form. Processed based on the data from original papers [2,3,15,16,17]. Notes: EA include ergocornine, ergocorninine, ergocristine, ergocristinine, ergocryptine, ergocryptinine, ergometrine, ergosine, ergosinine, ergotamine, and ergotaminine.

A total of 21 mycotoxins (3-AcDON, AFB_1_, AME, AOH, BEA, DAS, DON, ENNA, ENNA_1_, ENNB, ENNB_1_, FB_3_, FUS-X, HT-2, MPA, NEO, STEG, T-2, TEA, TEN, ZEA) have been found positive at least once in one of the forms throughout all five studies. On the contrary, a total of 36 mycotoxins (3/15-AcDON, AFB_2_, AFG_1_, AFG_2_, AGC, CIT, CPA, DON-3G, EA, FB_1_, FB_2_, GLI, MEL, NIV, OTA, PAT, PAX, PeA, PenA, PHO-A, ROC, STLAC, VER, VERR, α-ZOL, β-ZOL) have been tested in various MT-samples, but have never been confirmed positive. For more details regarding the positivity/negativity and the number of tested samples in the given categories see Figure 2. Among all mycotoxins, AME, AOH, BEA, DON, ENNA, ENNA_1_, ENNB, ENNB_1_, HT-2, T-2, TEN, and ZEA seem to be the most significant in MT-based dietary supplements. In this review, special attention will be given to these significant mycotoxins (see Section 8).

As can be seen in Figure 2, AFs (117 samples), AFB_1_ (68), OTA (67), DON (67), T-2 (67), ZEA (67), FB_1_ (65), FB_2_ (65), FUS-X (65), HT-2 (65), and STEG (65) are the most frequently analyzed mycotoxins in MT-based dietary supplements, followed by AME (58), AOH (58), BEA (58), DAS (58), ENNA (58), ENNA_1_ (58), ENNB (58), ENNB_1_ (58), FB_3_ (58), MPA (58), NEO (58), PAT (58), PenA (58), and TEN (58). Regarding the positivity of samples for a given mycotoxin, the frequency of testing should be taken into consideration as the percentages below are the more conclusive the more samples they are based on. For that reason, the categorization into seven levels: 1) Extremely high (more than 90%), 2) Very high (up to 90%), 3) High (up to 75%), 4) Moderate (up to 50%), 5) Low (up to25%), 6) Rare (up to 5%), and 7) None (0%) are based on data with at least 50 tested samples on a given mycotoxin. Extremely high positivity has been found in case of AME (98.28%, 57/58), ENNB_1_ (94.83%, 55/58), AOH (93.10%, 54/58), BEA (93.10%, 54/58), and ENNB (93.10%, 54/58), very high positivity in case of ENNA_1_ (89.66%, 52/58), ENNA (87.93%, 51/58), TEN (86.21%, 50/58), and T-2 (77.61%, 52/67), high positivity in case of HT-2 (73.85%, 48/65), ZEA (73.13%, 49/67), and DON (55.22%, 37/67), low positivity in case of NEO (18.97%, 11/58), AFs (15.38%, 18/117), DAS (6.90%, 4/58), MPA (6.90%, 4/58), and FUS-X (6.15%, 4/65), as rare in case of STEG (4.62%, 3/65), AFB_1_ (2.94%, 2/68), and FB_3_ (1.72%, 1/58), and none in case of OTA (0%, 0/67), FB_1_ (0%, 0/65), FB_2_ (0%, 0/65), PAT (0%, 0/58), and PenA (0%, 0/58).

### 7.1. Seeds

Beside seeds [2,15,16,17], the category *“Seeds”* also includes several samples of seeds intended for the preparation of tea [15,17]. A total of 31 mycotoxins have been analysed in MT seeds of which a total of 16 mycotoxins have been found positive. Compared to other categories, seeds appear to be relatively more contaminated with 3-AcDON, AFs, FUS-X, and NEO. For more details concerning the positivity of seed samples see Figure 2.

In seeds, the highest concentrations have reached up to 1900 µg/kg for AME, 1740 µg/kg for ENNB, 1450 µg/kg for AOH, 975 µg/kg for TEA [15], 943.7 µg/kg for HT-2 [2], 681 µg/kg for ENNB_1_ [15], 453.9 µg/kg for T-2 [2], 293 µg/kg for DON, 274 µg/kg for ENNA_1_, 265 µg/kg for 3-AcDON [15], 236.7 µg/kg for FBs [16], 234 µg/kg for BEA, 202 µg/kg for ENNA, 201 µg/kg for TEN, 199 µg/kg for FUS-X, 110 µg/kg for ZEA, 36 µg/kg for NEO [15], 11.5 µg/kg for AFs [16], 1.9 µg/kg for AFB_1_ [17].

### 7.2. Capsules

The category *“Capsules”* consists of capsules with dried powder [3,15], capsules with oil-based matrix [15], and encapsulated oily paste [3]. A total of 57 mycotoxins have been analysed in MT capsules of which a total of 20 mycotoxins have been found positive. Compared to other categories, capsules appear to be relatively more contaminated with DAS, DON, HT-2, MPA, and T-2. For more details concerning the positivity of capsule samples see Figure 2. 

The maximum levels have been reaching up to 10,940 µg/kg for ENNB_1_, 9260 µg/kg for ENNB, 8340 µg/kg for ENNA [15], 6834 µg/kg for AOH, 6477 µg/kg for DON, 5958 µg/kg for T-2, 3891 µg/kg for BEA [3], 3200 for AME [15], 2985 µg/kg for HT-2 [3], 2340 µg/kg for ENNA_1_, 2140 µg/kg for TEA [15], 2127 µg/kg for TEN [3], 1710 µg/kg for MPA, 751 µg/kg for ZEA, 175 µg/kg for 3-AcDON, 126 µg/kg for NEO, 120 µg/kg for FUS-X [15], 59 µg/kg for DAS [3], 13 µg/kg for FB_3_, and 11 µg/kg for STEG [15].

### 7.3. Tablets

A total of 25 mycotoxins have been analysed in MT-tablets of which a total of 13 mycotoxins have been found positive. Compared to other categories, tablets appear to be relatively more contaminated with TEA. For more details concerning the positivity of tablets samples see Figure 2.

The maximum levels have been reaching up to 2110 µg/kg for ENNB, 2020 µg/kg for AME, 1560 µg/kg for DON, 1370 µg/kg for TEA, 1340 µg/kg for AOH, 988 µg/kg for TEN, 842 µg/kg for BEA, 716 µg/kg for ENNB_1_, 640 µg/kg for T-2, 582 µg/kg for HT-2, 403 µg/kg for ZEA, 380 µg/kg for ENNA_1_, and 186 µg/kg for ENNA [15]. 

### 7.4. Granules

Only one sample of MT granules have been analysed for a total of 25 mycotoxins of which a total of 5 mycotoxins have been found positive with the following levels: 23 µg/kg for AOH, 16 µg/kg for ENNB, 6 µg/kg for ENNB_1_, 5 µg/kg for BEA, and 3 µg/kg for AME [15]. For more details concerning the positivity of granule samples see Figure 2.

### 7.5. Extracts

The category *“Extracts”* covers natural extract in glycerin [2], oil-based liquid seed extract and alcohol-based liquid seed extract [17]. A total of 16 mycotoxins have been analysed in MT extract of which a total of 2 mycotoxins have been found positive. The maximum levels have been up to 0.06 µg/kg for AFB_1_ (and AFs at the same time) [17]. For more details concerning the positivity of extract samples see Figure 2.

### 7.6. Herbs

So far, MT herbs (powdered or minced) have been analysed only for AFs and AFB_1_, but none of the tested samples has been found positive [17].

## 8. The Most Significant Mycotoxins in Milk Thistle-Based Dietary Supplements

**Figure 3:**
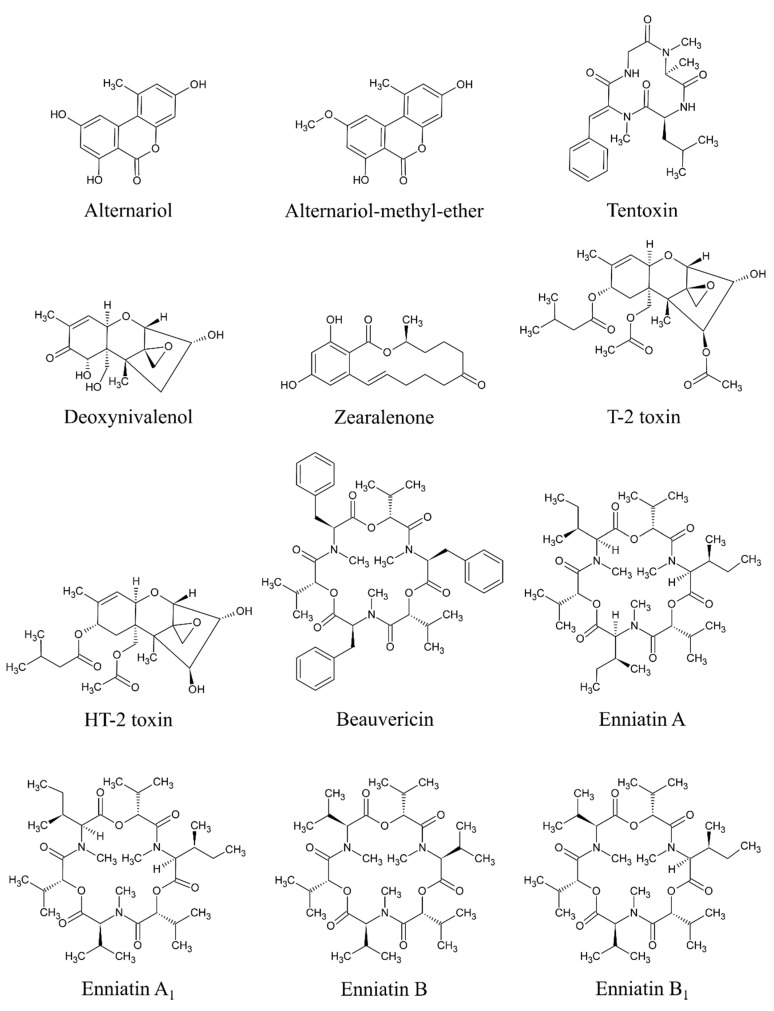
Chemical structures of significant mycotoxins found in milk thistle-based dietary supplements.

Based on available studies on the occurrence of mycotoxins in MT-based dietary supplements, the most critical mycotoxins appear to be AME, AOH, and TEN produced by *Alternaria* species and BEA, DON, ENNA, ENNA_1_, ENNB, ENNB_1_, HT-2, T-2, and ZEA produced by *Fusarium* species. All of these mycotoxins have shown an overall positivity of more than 50% (at least 55% in case of DON, up to 98% in case of AME) based on at least 58 samples. All of these 12 mycotoxins are considered significant in this study and will be given special attention (see below). The data regarding the positivity and concentrations of *“significant mycotoxins”* are based on original studies reviewed by Arroyo-Manzanares et al. [2], Fenclova et al. [3], Santos et al. [16], and Veprikova et al. [15]. The chemical structure of these significant mycotoxins is shown in Figure 3.

### 8.1. Alternaria Mycotoxins (AME, AOH, TEN)

*Alternaria* mycotoxins are produced by *Alternaria* genus [50], with *A. alternata* being the most common species [51,52]. However, *A. tenuissima, A. arborescens* [53], *A. tangelonis*, and *A. turkisafria* [54] are also significant in food. *Alternaria* fungi produce more than 70 different secondary metabolites [55]. Some of these are significant contaminants in food such as fruits, vegetables, cereals and derived products, and oilseeds [55]. AME (PubChem CID: 5360741), AOH (PubChem CID: 5359485), and TEN (PubChem CID: 5281143) [28] appear to be significant contaminants in MT-based supplements. Generally, AOH and AME are so far the most commonly studied *Alternaria* metabolites [56]. 

Among the *Alternaria* mycotoxins, hepatotoxic, genotoxic, mutagenic, clastogenic, immunotoxic and dermatoxic effects, reproductive toxicity, as well as an effect on estrogen activity, have been observed. Hepatotoxicity of AOH, AME and TEN have been suggested in vitro on the human hepatoma (HepaRG) cell line [57]. Genotoxicity of *Alternaria* toxin mixtures has been reported in vitro on human endometrial adenocarcinoma (Ishikawa) cells [56] and genotoxicity of AOH and AME has been reported on Chinese hamster (V79) cells, human liver (HepG2) cells and human colon (HT-29) cells [58]. Mutagenic effect of AOH has been observed in vitro on Chinese hamster (V79) cells and mouse lymphoma (L5178Y TK+/−) cells [59]. Clastogenic effect of AOH has been reported in vitro on human endometrial adenocarcinoma (Ishikawa) cells and Chinese hamster (V79) cells [60]. Immunotoxicity of AOH has been demonstrated in vitro on human colon adenocarcinoma (Caco-2) cells [61] or human monocytic (THP-1) cells [62]. Dermal toxicity of AOH has been demonstrated in vivo on mice [63]. Adverse effects on reproductive performance have been suggested in vitro on porcine ovarian cells [64]. The effect on estrogen activity has been reported in vitro and in silico on human endometrial adenocarcinoma (Ishikawa) cells and Chinese hamster (V79) cells [56,60,65]. Despite some esophageal carcinogenic effects of *Alternaria* mycotoxins (AOH and AME) having been reported [66], none has been classified by the International Agency for Research on Cancer (IARC) so far.

AME has proved to be the most common mycotoxin in MT-based supplements, occurring in 57 out of 58 total examined samples (7 seeds, 43 capsules, 6 tablets and 1 granule). The only negative sample was a tablet form.

The maximum levels of AME have been found in capsules containing dried powder (3200 µg/kg), followed by oil-based capsules (2110 µg/kg), tablets (2020 µg/kg) and seeds (1900 µg/kg) [15]. In the granule sample, a concentration of 3 µg/kg has been observed [15].

AOH is among the mycotoxins with extremely high positivity in MT-based supplements, with 54 positive samples out of 58 total examined samples (6 out of 7 seed samples, 41 out of 43 capsules, 6 out of 7 tablets and 1 out of 1 granule).

The maximum levels of AOH have been found in capsules containing dried powder (6834 µg/kg), followed by oil-based capsules (1964 µg/kg) [3], seeds (1450 µg/kg) and tablets (1340 µg/kg) [15]. In the granule sample, a concentration of 23 µg/kg has been observed [15].

Although less significant than AME and AOH, the positivity of TEN in MT-based supplements is still very high: 50 positive samples out of 58 total examined samples (5 out of 7 seed samples, 39 out of 43 capsules, 6 out of 7 tablets, and 0 out of 1 granule).

The maximum levels of TEN have been found in capsules containing dried powder (2127 µg/kg) [3], followed by tablets (988 µg/kg), oil-based capsules (772 µg/kg) and seeds (201 µg/kg) [15].

### 8.2. Fusarium Mycotoxins

Four “common” *Fusarium* mycotoxins occur in MT-based supplements in significant amounts –DON (PubChem CID: 40024), T-2 (PubChem CID: 5759), HT-2 (PubChem CID: 520286), ZEA (PubChem CID: 5281576) [28]. Moreover, some emergent *Fusarium* mycotoxins–BEA (PubChem CID: 3007984), ENNA (PubChem CID: 57339252), ENNA_1_ (PubChem CID: 57339253), ENNB (PubChem CID: 164754), and ENNB_1_ (PubChem CID: 11262300) [28] are also significant.

### 8.3. Trichothecenes (DON, T-2, HT-2)

Trichothecenes (TCT) are a group of chemically related mycotoxins (types A-D). In food, TCT are produced by the *Fusarium* genera. T-2/HT-2 (type A) and DON (type B) are significant contaminants of MT. DON is the most important TCT produced mainly by *F. graminearum* and *F. culmorum*, especially in cereals [67]. T2/HT-2 are produced mainly by *F. sporotrichioides*, *F*. *landsethiae*, *F. poae*, and *F. sambucinum* [67].

Cytotoxic, hepatotoxic, neurotoxic, and immunotoxic effects, as well as reproductive toxicity and skin toxicity, have been reported for both T-2 and DON. In vivo hepatotoxic effects have been reported on mice in case of DON [68] and on broilers in case of T-2 [69]. Neurotoxic effects in vivo have been reported on chicks in case of DON [70] and on rats in case of T-2 [71]. The immunotoxic effect of T-2 has been reported on rainbow trout (*Oncorhynchus mykiss*) in vivo [72] and the cytotoxic effect on monocytes, macrophages, dendritic cells and B and T lymphocytes in vitro [73,74,75]. DON was reported to be less cytotoxic on dendritic cells in vitro than T-2 [76]. Reproductive toxicity has been reported on male mice in vivo in case of T-2 [77] and on boar semen in vitro in case of DON [78]. Skin toxicity has been demonstrated for T-2 on mice and rabbits in vivo [79,80] and suggested for DON in vitro on human immortalized keratinocytes [81]. Moreover, in vitro, the cytotoxic effect of T-2 and DON on human liver cancer (HepG2) cells has been confirmed [82,83]. T-2(/HT-2)-induced cytotoxicity on human chondrocytes [84] and broiler hepatocytes [85] in vitro has been reported. In terms of carcinogenicity, T-2 and DON are classified by the IARC into group 3 “Not classifiable as to its carcinogenicity to humans” [86], but no data are available on the carcinogenicity of HT-2 [87].

DON is the least occurring among the significant mycotoxins in MT-based dietary supplements, as it has been found only in 37 out of 67 total examined samples (4 out of 15 seed samples, 29 out of 43 capsules, 4 out of 7 tablets, 0 out of 1 granule, and 0 out of 1 extract). 

The maximum levels of DON have been found in capsules containing dried powder (6477 µg/kg) [3], followed by oil-based capsules (2890 µg/kg), tablets (1560 µg/kg), and seeds (293 µg/kg) [15].

T-2 has been found in 52 out of 67 total examined samples (10 out of 15 seed samples, 38 out of 43 capsules, 4 out of 7 tablets, 0 out of 1 granule, and 0 out of 1 extract). The maximum levels of T-2 have been found in capsules with dried powder (5958 µg/kg) [3], followed by oil-based capsules (1870 µg/kg), tablets (640 µg/kg) [15], and seeds (453.9 µg/kg) [2].

HT-2 has been found positive in 48 out of 65 total examined samples (7 out of 13 seed samples, 38 out of 43 capsules, 3 out of 7 tablets, 0 out of 1 granule, and 0 out of 1 extract). The maximum levels of HT-2 have been found in capsules with dried powder (2985 µg/kg) [3], followed by oil-based capsules (1530 µg/kg) [15], seeds (943.7 µg/kg) [2], and tablets (582 µg/kg) [15].

### 8.4. Zearalenone (ZEA)

ZEA is a non-steroidal estrogenic mycotoxin produced mainly by the *Fusarium* genera [88]. *F. graminearum* and *F. culmorum* are the main ZEA producers in food. *F. equiseti* and *F. crookwellense* also produce ZEA [67]. ZEA is a common contaminant in grains, mainly in maize, but also in other cereals such as wheat, barley, oat and sorghum [89,90]. Nevertheless, in the context of this review, ZEA has been shown to be a significant contaminant in MT-supplements.

ZEA is often associated with reproductive disorders in livestock (e.g., pigs, cattle, and sheep) and occasionally exerts hyper-estrogenic syndrome in humans [91]. Recently, ZEA reproductive toxicity has been demonstrated in vitro on boar semen [78,92] and in vivo on rats [42], or model organism *Artemia francisacana* [93]. The estrogenic effect has been observed in vitro on human endometrial cancer (Ishikawa) cells [94]. Moreover, developmental toxicity and fetotoxicity have been reported on mice in vivo [95] and embryotoxicity has been observed in vitro on early porcine embryos [96] and human embryonic stem cells (hESC) [97]. 

Besides reproductive and developmental toxicity, xenoestrogenity, fetotoxicity and embryotoxicity, ZEA was reported to exert cytotoxic, cardiotoxic, nephrotoxic, hepatotoxic, immunotoxic, genotoxic and neurotoxic effects. ZEA-induced cardiotoxicity has been reported in vivo on mice [98]. The nephrotoxicity of ZEA has been reported in vivo on rats [99,100]. The hepatotoxic effect was observed in vitro on rats [100] and mice [68]. The immunotoxicity of ZEA has been confirmed on mice [101] and rats [102] in vivo and suggested in vitro on swine spleen [103]. ZEA has been found to promote apoptosis, autophagy and DNA damage in porcine blastocysts [96]. The cytotoxic effect of ZEA has been demonstrated in vitro on human liver cancer (HepG2) cells [82,104,105], human adrenocortical carcinoma (H295R) cells [106], murine Leukemia virus-induced tumor (RAW 264.7) cells [82] and pig intestinal epithelial (IPEC-J2) cells [107]. ZEA has been reported to affect mouse brain function in vivo [108]. Recent studies confirm a gastro-toxic effect of ZEA on piglets [109] and rats [110] in vivo and reveal in vitro gastro-toxic effects on porcine jejunum explant [111]. From the point of view of the carcinogenicity, ZEA has been classified by IARC into group 3 “Not classifiable as to its carcinogenicity to humans” [86].

ZEA has been found in 49 out of 67 total examined samples (7 out of 15 seed samples, 37 out of 43 capsules, 5 out of 7 tablets, 0 out of 1 granule, and 0 out of 1 extract). The maximum levels of ZEA have been found in capsules with dried powder (751 µg/kg), followed by tablets (403 µg/kg), oil-based capsules (373 µg/kg), and seeds (110 µg/kg) [15].

### 8.5. Emergent Mycotoxins (BEA, ENNs)

ENNs and BEA are considered emergent in the recent literature [112]. They are non-trichothecene secondary metabolites produced by the *Fusarium* species in particular [113,114,115]. In food, they are both produced by *Fusarium acuminatum, F. avenaceum*, *F. poae*, *F. sambucinum* and *F. sporotrichioides*. The other BEA food-born producers are *F. dalminii, F*. *equiseti*, *F. longipes*, *F. nygamai, F. oxysporum, F.proliferatum, F. subglutinans*, *F. verticillioides*. The other ENN food-borne producers are *F. langsethiae* and *F. lateritium* [67]. 

The European Food Safety Authority (EFSA) has concluded that neither BEA nor ENNs indicates a serious problem for human health in acute exposure [114], which may be relevant to their rapid absorption, distribution and elimination [116]. The cytotoxic effects in vitro of both BEA and ENNs are widely researched and confirmed by many studies. Their cytotoxic effect has been reported on human colon adenocarcinoma (Caco-2) cells [117,118,119], human liver cancer (HepG2) cells, human bronchial (BEAS-2B) cells, human gastric (N87) cells, human vascular endothelial cells (HUVEC), and human keratinocytes (HEK) [119]. Moreover, BEA has been reported cytotoxic in human neuroblastoma (SH-SY5Y) cells [120], while ENNs have shown cytotoxic effects on human cervix carcinoma (HeLa) cells (ENNA) [121]. In some cases, both BEA and ENNs (namely ENNA) have been reported to have a mild genotoxic [117,121] or hemolytic [119] effect. In addition, ENNB_1_ has been reported to induce oxidative stress and immunotoxic effects during mouse embryo development [122]. A recent in vivo study showed an overall toxic effect of BEA on *Caenorhaditis elegans*, reducing its life span and exerting reproductive and developmental toxicity, cyto-toxicity and oxidative stress [123].

Due to the common producers, as well as the similar chemical structure of these mycotoxins, their co-occurrence can be expected. However, they can also occur together with other *Fusarium* mycotoxins [114]. Although they are considered to occur especially in cereal grains and grain-based products [114], they have also been shown as significant in this review concerning MT-based supplements.

BEA has been found in 54 out of 58 total examined samples (6 out of 7 seed samples, 41 out of 43 capsules, 6 out of 7 tablets, and 1 out of 1 granule). The maximum levels of BEA have been found in capsules with dried powder (3891 µg/kg) [3], followed by oil-based capsule (1560 µg/kg), tablets (842 µg/kg), seeds (234 µg/kg), and granules (5 µg/kg) [15].

ENNA has been found in 51 out of 58 total examined samples (5 out of 7 seed samples, 40 out of 43 capsules, 6 out of 7 tablets, 0 out of 1 granule). The maximum levels have been found in oil-based capsules (8340 µg/kg), followed by capsules with dried powder (4240 µg/kg), seeds (202 µg/kg), and tablets (186 µg/kg) [15].

ENNA_1_ has been found in 52 out of 58 total examined samples (6 out of 7 seed samples, 40 out of 43 capsules, 6 out of 7 tablets, an 0 out of 1 granule). The maximum levels have been found in oil-based capsules (2340 µg/kg), followed by capsules with dried powder (1420 µg/kg), tablets (380 µg/kg), and seeds (274 µg/kg) [15].

ENNB has been found in 54 out of 58 total examined samples (6 out of 7 seed samples, 41 out of 43 capsules, 6 out of 7 tablets, and 1 out of 1 granule). The maximum levels have been found in oil-based capsules (9260 µg/kg), followed by capsules with dried powder (6190 µg/kg), tablets (2110 µg/kg), seeds (1740 µg/kg), and granules (16 µg/kg) [15].

ENNB_1_ has been found in 55 out of 58 total examined samples (6 out of 7 seed samples, 42 out of 43 capsules, 6 out of 7 tablets). The maximum levels have been found in capsules with dried powder (10,940 µg/kg), followed by oil-based capsules (4750 µg/kg), tablets (716 µg/kg), seeds (681 µg/kg), and granules (6 µg/kg) [15].

## 9. Mycotoxin Regulations

The presence of mycotoxins in herbal-based food supplements cannot be completely avoided. There is a need to establish maximum levels or action levels of mycotoxins in some kinds of commodities. Risk management is significantly applied here. No regulatory limits for herbal-based food supplements have been incorporated into legislation so far. The maximum regulatory limits for certain mycotoxins in foods have been set under EU regulation No. 1881/2006 [124], and later decrees as in force. Nevertheless, in the case of herbs, the legislation covers only AFs and OTA. The maximum limits of 5 and 10 µg/kg for AFB_1_ and sum of AFs, respectively, have been set for ginger [124]. For OTA, the maximum limit of 20 µg/kg has been set for liquorice root, ingredient for herbal infusion and 80 µg/kg for liquorice extract, for use in food in particular beverages and confectionary [125]. 

## 10. Mycotoxin Exposure Assessment and Risk Characterization

The Joint FAO/WHO Expert Committee on Food Additives (JEFCA) established provisional maximum tolerable daily intakes (PMTDI) for DON and its acetylated derivates (3-AcDON and 15-AcDON) of 1 µg/kg body weight (bw) per day [126]. A current group tolerable daily intake (TDI) of 1 µg/kg bw was established for the sum of DON, 3-AcDON, 15-AcDON and DON-3G, based on reduced body weight gain in experimental female and male mice [127].

PMTDI was established for T-2 and HT-2 alone or in combination of 0.06 µg/kg bw per day obtained in a 3-week dietary study in pigs [128]. A new group TDI of 0.02 µg/kg bw was established by EFSA for the sum of T-2 and HT-2 based on an in vivo sub-chronic toxicity study with rats [129].

PMTDI was established for ZEA of 0.5 µg/kg bw based on the no observed effect level (NOEL) of 40 µg/kg bw per day obtained in a 15-day study in pigs [130]. The current TDI for ZEA of 0.25 µg/kg bw per day established by EFSA is based on estrogenicity in pigs [131].

The increased incidence of microscopic kidney lesions seen in a 3-month feeding study with pigs [132] was considered as the most appropriate endpoint of non-neoplastic effects of OTA and the resulting benchmark dose limit (BMDL) of 4.73 µg/kg bw per day was used for comparison with chronic exposures.

In the absence of elucidated MoAs for the genotoxicity/carcinogenicity of OTA, the Panel concluded that a margin of exposure (MOE) of 10,000 needs to be applied to the BMDL_10_ of 14.5 µg/kg bw per day for neoplastic effects (kidney tumors) in the rat. The Panel points out that this MOE is likely to be particularly conservative in this case, as the evidence for a direct interaction of OTA with the DNA is inconclusive and other threshold mechanisms may play a role in the formation of kidney tumors. As it was not possible to quantify these variables, the default MOE of 10,000 was applied [133].

Based on studies in animals, the CONTAM Panel selected a BMDL_10_ of 0.4 µg/kg bw per day for the incidence of hepatocellular carcinoma (HCC) in male rats following AFB_1_ exposure to be used in a MOE approach. The calculation of a BMDL from the human data was not appropriate; instead, the cancer potencies estimated by the JECFA in 2016 were used [134].

Studies evaluating the dietary exposure to mycotoxins from MT food supplements are scarce. Several studies have attempted a very rough assessment of dietary exposure based on the RDD of food supplements (e.g., capsules) declared by the manufacturers.

For DON, TDI has been set at 1 μg/kg bw per day [135], which means 70 μg for a 70 kg human. In the worst-case scenario, for a human of this weight, a single RDD of 3 capsules of MT-based supplement has accounted for 23.0% of TDI [3]. On average, 2.1% of TDI is received by the MT-based supplements [3,15].

For ZEA, the TDI has been set at 0.25 μg/kg per day [136], which means 17.5 μg for a 70 kg human. In the worst-case scenario, for a human of this weight, a single RDD of 10 capsules of MT-based supplement has accounted for 5.3% of TDI [15]. On average, 1.0% of TDI is received by the MT-based supplements [3,15].

For the sum of T-2 and HT-2, the TDI has been set at 0.02 μg/kg bw per day [129], which means 1.4 μg for a 70 kg human. In the worst-case scenario, for a human of this weight, a single RDD of 3 capsules of MT-based supplement has accounted for 1590% of TDI [3]. On average, 123% of TDI is received by the MT-based supplements [3,15].

There is insufficient data to establish dietary exposure assessment for any *Alternaria* mycotoxins [137], ENNs or BEA [114]. 

## 11. Summary

People use silymarin preparations to prevent or treat various diseases, especially, but not limited to, liver diseases. Although silymarin appears to be effective in this aspect, a number of various mycotoxins with, inter alia, hepatotoxic effects have been found in marketed MT preparations. Studies have shown that silymarin can alleviate the adverse effects of some mycotoxins, notably AFB_1_, but also OTA, FB_1_, ZEA or DON. However, the latter two have also been shown to occur in MT-based supplements to a considerable extent. In addition, it has been shown that the content of silymarin in the preparations varies considerably. The question arises as to whether the consumption of these supplements in order to improve health does not become rather harmful, with a regard to the detected levels of mycotoxins. It is, therefore, necessary to monitor both the content of active compounds in MT-based supplements and the presence of mycotoxins and other contaminants, to assess the intake of the substances into the body, and to evaluate whether the beneficial effects of marketed MT-preparations outweigh the harmful effects of the contaminants. It should also be borne in mind that people may take more than one type of food supplement at the same time, which is worrying if the other supplements are also contaminated with mycotoxins to a similar extent.

This review examined the current state of contamination of MT-based dietary supplements with mycotoxins and, to a lesser extent, micro-fungi. The results show that these supplements are mainly infested by micro-fungi of the *Alternaria* genus. Of the 57 mycotoxins monitored across five original studies concerning MT-based supplements in various forms, a total of 21 have been found to be positive in at least one case. A total of 12 (AME, AOH, TEN, DON, HT-2, T-2, ZEA BEA, ENNA, ENNA_1_, ENNB, ENNB_1_) of these mycotoxins can be considered significant due to their high occurrence meaning more than 50% of positive samples in the context of this review.

The obtained overview strongly indicates the need for the strict monitoring of mycotoxins in commercially sold MT-based dietary supplements that are used by many people worldwide to treat or prevent liver diseases, and thereby enhance their health.

## Figures and Tables

**Table 1:** Overview of the methods used in studies dealing with mycotoxins in milk thistle-based dietary supplements.

Supplement Form	Mycotoxins	Clean-up Method	Analysis	References
Seeds	7 mycotoxins	multifunctional columns (for AFs, ZEA, DON, FBs, T-2); polyamide column (for CIT); no clean-up (for OTA)	ELISA	[16]
Seeds, herbs, tea, alcohol-based liquid seed extract, oil-based liquid seed extract	AFs, AFB_1_	immunoaffinity column clean-up	RPLC-FLD	[17]
Seeds, extract	15 mycotoxins	QuEChERS + DLLME (for AFB_1_, AFB_2_, AFG_1_, AFG_2_, CIT, HT-2, OTA, STEG, T-2, ZEA)	UHPLC-MS/MS	[2]
Capsules with dried powder/oil-based matrix, seeds, tablets, granules, tea	57 mycotoxins	QuEChERS	UHPLC-MS/MS	[15]
Encapsulated oily paste, capsules with dried powder	55 mycotoxins	QuEChERS	UHPLC-HRMS	[3]

Notes: AFs, aflatoxins; AFB_1_, aflatoxin B_1_; AFB_2_, aflatoxin B_2_; AFG_1_, aflatoxin G_1_; AFG_2_, aflatoxin G_2_; CIT, citrinin; DON, deoxynivalenol; FBs, fumonisins; HT-2, HT-2 toxin; OTA, ochratoxin A; STEG, sterigmatocystin; T-2, T-2 toxin; ZEA, zearalenone; DLLME, dispersive liquid-liquid microextraction; ELISA, enzyme-linked immunosorbent assay; QuEChERS, quick easy cheap effective rugged safe; RPLC-FLD, reversed-phase liquid chromatography with fluorescence detector; UHPLC-HRMS, ultra-high-performance liquid chromatography-high-resolution mass spectrometry; UHPLC-MS/MS, ultra-high-performance liquid chromatography-mass spectrometry.

## References

[1] European Parliament and the Council of the European Union (2002). Directive 2002/46/EC of the European Parliament and of the Council of 10 June 2002 on the approximation of the laws of the member states relating to food supplements. Off. J. Eur. Communities.

[2] Arroyo-Manzanares N., García-Campaña A.M., Gámiz-Gracia L. (2013). Multiclass mycotoxin analysis in *Silybum marianum* by ultra high performance liquid chromatography–tandem mass spectrometry using a procedure based on QuEChERS and dispersive liquid–liquid microextraction. J. Chromatogr. A.

[3] Fenclova M., Novakova A., Viktorova J., Jonatova P., Dzuman Z., Ruml T., Kren V., Hajslova J., Vitek L., Stranska-Zachariasova M. (2019). Poor chemical and microbiological quality of the commercial milk thistle-based dietary supplements may account for their reported unsatisfactory and non-reproducible clinical outcomes. Sci. Rep..

[4] Fibigr J., Šatínský D., Solich P. (2018). Current trends in the analysis and quality control of food supplements based on plant extracts. Anal. Chim. Acta.

[5] Seeff L.B., Bonkovsky H.L., Navarro V.J., Wang G. (2015). Herbal products and the liver: A review of adverse effects and mechanisms. Gastroenterology.

[6] Ashiq S., Hussain M., Ahmad B. (2014). Natural occurrence of mycotoxins in medicinal plants: A review. Fungal Genet. Biol..

[7] Mavungu J.D.D., Monbaliu S., Scippo M.-L., Maghuin-Rogister G., Schneider Y.-J., Larondelle Y., Callebaut A., Robbens J., Peteghem C.V., Saeger S.D. (2009). LC-MS/MS multi-analyte method for mycotoxin determination in food supplements. Food Addit. Contam. Part A.

[8] Smith T., Gillespie M., Eckl V., Knepper J., Reynolds C.M. (2019). Herbal supplement sales in US increase by 9.4% in 2018. HerbalGram.

[9] Abenavoli L., Capasso R., Milic N., Capasso F. (2010). Milk thistle in liver diseases: Past, present, future. Phytother. Res..

[10] Asrani S.K., Devarbhavi H., Eaton J., Kamath P.S. (2019). Burden of liver diseases in the world. J. Hepatol..

[11] Cwalina-Ambroziak B., Wierzbowska J., Damszel M., Bowszys T. (2012). The effect of mineral fertilization on achenes yield and fungal communities isolated from the stems of milk thistle *Silybum marianum* (L.) Gaertner. Acta Sci. Pol. Hortorum Cultus.

[12] Rosińska A., Dorna H., Szopińska D., Seidler-Łożykowska K. (2017). Experimental paper. The effect of colour grading of milk thistle (*Silybum marianum* (L.) Gaertn.) seeds on their quality for sowing. Herba Pol..

[13] Rosińska A., Dorna H., Szopińska D., Irzykowska L., Seidler-Łożykowska K. (2018). Evaluation of milk thistle (*Silybum marianum* (L.) Gaertn.) seed germination in relation to seed health and seedling emergence. Herba Pol..

[14] Tournas V.H., Calo J.R., Sapp C. (2013). Fungal profiles in various milk thistle botanicals from US retail. Int. J. Food Microbiol..

[15] Veprikova Z., Zachariasova M., Dzuman Z., Zachariasova A., Fenclova M., Slavikova P., Vaclavikova M., Mastovska K., Hengst D., Hajslova J. (2015). Mycotoxins in plant-based dietary supplements: Hidden health risk for consumers. J. Agric. Food Chem..

[16] Santos L., Marín S., Sanchis V., Ramos A.J. (2009). Screening of mycotoxin multicontamination in medicinal and aromatic herbs sampled in Spain. J. Sci. Food Agric..

[17] Tournas V.H., Sapp C., Trucksess M.W. (2012). Occurrence of aflatoxins in milk thistle herbal supplements. Food Addit. Contam. Part A.

[18] Capriotti A.L., Caruso G., Cavaliere C., Foglia P., Samperi R., Laganà A. (2012). Multiclass mycotoxin analysis in food, environmental and biological matrices with chromatography/mass spectrometry. Mass Spectrom. Rev..

[19] Steyn P.S. (1995). Mycotoxins, general view, chemistry and structure. Toxicol. Lett..

[20] Wianowska D., Wiśniewski M. (2015). Simplified procedure of silymarin extraction from *Silybum marianum* L. Gaertner. J. Chromatogr. Sci..

[21] Andrzejewska J., Martinelli T., Sadowska K. (2015). *Silybum marianum*: Non-medical exploitation of the species. Ann. Appl. Biol..

[22] Karkanis A., Bilalis D., Efthimiadou A. (2011). Cultivation of milk thistle (*Silybum marianum* L. Gaertn.), a medicinal weed. Ind. Crops Prod..

[23] Bijak M. (2017). Silybin, a major bioactive component of milk thistle (*Silybum marianum* L. Gaernt.)—Chemistry, bioavailability, and metabolism. Molecules.

[24] Gresta F., Avola G., Guarnaccia P. (2006). Agronomic characterization of some spontaneous genotypes of milk thistle (*Silybum marianum* L. Gaertn.) in Mediterranean environment. J. Herbs Spices Med. Plants.

[25] Abenavoli L., Izzo A.A., Milić N., Cicala C., Santini A., Capasso R. (2018). Milk thistle (*Silybum marianum*): A concise overview on its chemistry, pharmacological, and nutraceutical uses in liver diseases. Phytother. Res..

[26] Fibigr J., Šatínský D., Solich P. (2017). A new approach to the rapid separation of isomeric compounds in a *Silybum marianum* extract using UHPLC core-shell column with F5 stationary phase. J. Pharm. Biomed. Anal..

[27] Javed S., Kohli K., Ali M. (2011). Reassessing bioavailability of silymarin. Altern. Med. Rev. J. Clin. Ther..

[28] PubChem https://pubchem.ncbi.nlm.nih.gov/.

[29] Mitchell S.T., Olson K.R. (2012). Chapter 231. Silymarin or Milk Thistle (*Silybum Marianum*). Poisoning & Drug Overdose.

[30] Fanoudi S., Alavi M.S., Karimi G., Hosseinzadeh H. (2020). Milk thistle (*Silybum Marianum*) as an antidote or a protective agent against natural or chemical toxicities: A review. Drug Chem. Toxicol..

[31] Aboelwafa H.R., El-kott A.F., Abd-Ella E.M., Yousef H.N. (2020). The possible neuroprotective effect of silymarin against aluminum chloride-prompted Alzheimer’s-like disease in Rats. Brain Sci..

[32] Guo H., Cao H., Cui X., Zheng W., Wang S., Yu J., Chen Z. (2019). Silymarin’s inhibition and treatment effects for Alzheimer’s disease. Molecules.

[33] El-Ashmawy N.E., Khedr E.G., El-Bahrawy H.A., Helmy N.N. (2020). Modulatory effect of silymarin on apoptosis in testosterone -induced benign prostatic hyperplasia in rats. Pathol. Oncol. Res..

[34] Saberi Z., Gorji N., Memariani Z., Moeini R., Shirafkan H., Amiri M. (2020). Evaluation of the effect of *Silybum Marianum* extract on menopausal symptoms: A randomized, double-blind placebo-controlled trial. Phytother. Res..

[35] Othman S., Ali S.M., Deeb N.M.E. (2020). Protective effect of *Silybum marianum* extract against doxorubicin induced toxicity in male rats. PSM Biol. Res..

[36] Rašković A., Stilinović N., Kolarović J., Vasović V., Vukmirović S., Mikov M. (2011). The protective effects of silymarin against doxorubicin-induced cardiotoxicity and hepatotoxicity in rats. Molecules.

[37] Vostálová J., Tinková E., Biedermann D., Kosina P., Ulrichová J., Rajnochová Svobodová A. (2019). Skin protective activity of silymarin and its flavonolignans. Molecules.

[38] Fidrus E., Ujhelyi Z., Fehér P., Hegedűs C., Janka E.A., Paragh G., Vasas G., Bácskay I., Remenyik É. (2019). Silymarin: Friend or foe of UV exposed keratinocytes?. Molecules.

[39] Alhidary I.A., Rehman Z., Khan R.U., Tahir M. (2017). Anti-aflatoxin activities of milk thistle (*Silybum marianum*) in broiler. Worlds Poult. Sci. J..

[40] Stoev S.D., Njobeh P., Zarkov I., Mircheva T., Zapryanova D., Denev S., Dimitrova B. (2019). Selected herbal feed additives showing protective effects against ochratoxin A toxicosis in broiler chicks. World Mycotoxin J..

[41] Ledur P.C., Santurio J.M. (2020). Cytoprotective effects of curcumin and silymarin on PK-15 cells exposed to ochratoxin A, fumonisin B1 and deoxynivalenol. Toxicon.

[42] Gao X., Xiao Z.-H., Liu M., Zhang N.-Y., Khalil M.M., Gu C.-Q., Qi D.-S., Sun L.-H. (2018). Dietary silymarin supplementation alleviates zearalenone-induced hepatotoxicity and reproductive toxicity in rats. J. Nutr..

[43] Gillessen A., Schmidt H.H.-J. (2020). Silymarin as supportive treatment in liver diseases: A narrative review. Adv. Ther..

[44] Grela E.R., Świątkiewicz M., Florek M., Wojtaszewska I. (2020). Impact of milk thistle (*Silybum marianum* L.) seeds in fattener diets on pig performance and carcass traits and fatty acid profile and cholesterol of meat, backfat and liver. Livest. Sci..

[45] Kosina P., Dokoupilová A., Janda K., Sládková K., Silberová P., Pivodová V., Ulrichová J. (2017). Effect of *Silybum marianum* fruit constituents on the health status of rabbits in repeated 42-day fattening experiment. Anim. Feed Sci. Technol..

[46] Tedesco D., Tava A., Galletti S., Tameni M., Varisco G., Costa A., Steidler S. (2004). Effects of silymarin, a natural hepatoprotector, in periparturient Dairy Cows. J. Dairy Sci..

[47] Khamisabadi H. (2020). Effects of Silymarin on milk production, liver enzymes, oxidative status and HSP70 gene expression in postparturient Sanjabi ewes. Cell. Mol. Biol..

[48] Šťastník O., Mrkvicová E., Pavlata L., Roztočilová A., Umlášková B., Anzenbacherová E. (2019). Performance, biochemical profile and antioxidant activity of hens supplemented with addition of milk thistle (*Silybum marianum*) seed cakes in diet. Acta Univ. Agric. Silvic. Mendel. Brun..

[49] World Health Organization (2007). WHO Guidelines for Assessing Quality of Herbal Medicines with Reference to Contaminants and Residues.

[50] Ostry V. (2008). *Alternaria* mycotoxins: An overview of chemical characterization, producers, toxicity, analysis and occurrence in foodstuffs. World Mycotoxin J..

[51] Logrieco A., Bottalico A., Mulé G., Moretti A., Perrone G. (2003). Epidemiology of toxigenic fungi and their associated mycotoxins for some mediterranean Crops. Eur. J. Plant Pathol..

[52] Romero S.M., Comerio R.M., Larumbe G., Ritieni A., Vaamonde G., Fernández Pinto V. (2005). Toxigenic fungi isolated from dried vine fruits in Argentina. Int. J. Food Microbiol..

[53] Andersen B., Krøger E., Roberts R.G. (2002). Chemical and morphological segregation of *Alternaria arborescens*, *A. infectoria* and *A. tenuissima* species-groups. Mycol. Res..

[54] Andersen B., Hansen M.E., Smedsgaard J. (2005). Automated and unbiased image analyses as tools in phenotypic classification of small-spored *Alternaria* spp.. Phytopathology.

[55] European Food Safety Authority (2016). Dietary exposure assessment to *Alternaria* toxins in the European population. EFSA J..

[56] Aichinger G., Krüger F., Puntscher H., Preindl K., Warth B., Marko D. (2019). Naturally occurring mixtures of *Alternaria* toxins: Anti-estrogenic and genotoxic effects in vitro. Arch. Toxicol..

[57] Hessel-Pras S., Kieshauer J., Roenn G., Luckert C., Braeuning A., Lampen A. (2019). In vitro characterization of hepatic toxicity of *Alternaria* toxins. Mycotoxin Res..

[58] Pfeiffer E., Eschbach S., Metzler M. (2007). *Alternaria* toxins: DNA strand-breaking activity in mammalian cells in vitro. Mycotoxin Res..

[59] Brugger E.-M., Wagner J., Schumacher D.M., Koch K., Podlech J., Metzler M., Lehmann L. (2006). Mutagenicity of the mycotoxin alternariol in cultured mammalian cells. Toxicol. Lett..

[60] Lehmann L., Wagner J., Metzler M. (2006). Estrogenic and clastogenic potential of the mycotoxin alternariol in cultured mammalian cells. Food Chem. Toxicol..

[61] Schmutz C., Cenk E., Marko D. (2019). The *Alternaria* mycotoxin alternariol triggers the immune response of IL-1β-stimulated, differentiated Caco-2 cells. Mol. Nutr. Food Res..

[62] Kollarova J., Cenk E., Schmutz C., Marko D. (2018). The mycotoxin alternariol suppresses lipopolysaccharide-induced inflammation in THP-1 derived macrophages targeting the NF-κB signalling pathway. Arch. Toxicol..

[63] Bansal M., Singh N., Alam S., Pal S., Satyanarayana G.N.V., Singh D., Ansari K.M. (2019). Alternariol induced proliferation in primary mouse keratinocytes and inflammation in mouse skin is regulated via PGE2/EP2/cAMP/p-CREB signaling pathway. Toxicology.

[64] Tiemann U., Tomek W., Schneider F., Müller M., Pöhland R., Vanselow J. (2009). The mycotoxins alternariol and alternariol methyl ether negatively affect progesterone synthesis in porcine granulosa cells in vitro. Toxicol. Lett..

[65] Dellafiora L., Warth B., Schmidt V., Del Favero G., Mikula H., Fröhlich J., Marko D. (2018). An integrated in silico/in vitro approach to assess the xenoestrogenic potential of *Alternaria* mycotoxins and metabolites. Food Chem..

[66] Liu G.T., Qian Y.Z., Zhang P.E., Dong W.H., Qi Y.M., Guo H. (1992). Etiological role of *Alternaria alternata* in human esophageal cancer. Chin. Med. J..

[67] Frisvad J.C., Thrane U., Samson R.A., Dijksterhuis J., Samson R.A. (2007). Mycotoxin producers. Food Mycology: A Multifaceted Approach to Fungi and Food.

[68] Sun L.-H., Lei M., Zhang N.-Y., Zhao L., Krumm C.S., Qi D.-S. (2014). Hepatotoxic effects of mycotoxin combinations in mice. Food Chem. Toxicol..

[69] Yin H., Han S., Chen Y., Wang Y., Li D., Zhu Q. (2020). T-2 Toxin induces oxidative stress, apoptosis and cytoprotective autophagy in chicken hepatocytes. Toxins.

[70] Wang X., Tang J., Geng F., Zhu L., Chu X., Zhang Y., Rahman S.U., Chen X., Jiang Y., Zhu D. (2018). Effects of deoxynivalenol exposure on cerebral lipid peroxidation, neurotransmitter and calcium homeostasis of chicks in vivo. Toxicon.

[71] Guo P., Liu A., Huang D., Wu Q., Fatima Z., Tao Y., Cheng G., Wang X., Yuan Z. (2018). Brain damage and neurological symptoms induced by T-2 toxin in rat brain. Toxicol. Lett..

[72] Modra H., Palikova M., Hyrsl P., Bartonkova J., Papezikova I., Svobodova Z., Blahova J., Mares J. (2020). Effects of trichothecene mycotoxin T-2 toxin on haematological and immunological parameters of rainbow trout (*Oncorhynchus mykiss*). Mycotoxin Res..

[73] Hymery N., Léon K., Carpentier F.-G., Jung J.-L., Parent-Massin D. (2009). T-2 toxin inhibits the differentiation of human monocytes into dendritic cells and macrophages. Toxicol. In Vitro.

[74] Vlata Z., Porichis F., Tzanakakis G., Tsatsakis A., Krambovitis E. (2005). In vitro cytopathic effects of mycotoxin T-2 on human peripheral blood T lymphocytes. Toxicol. Lett..

[75] Minervini F., Fornelli F., Lucivero G., Romano C., Visconti A. (2005). T-2 toxin immunotoxicity on human B and T lymphoid cell lines. Toxicology.

[76] Hymery N., Sibiril Y., Parent-Massin D. (2006). In vitro effects of trichothecenes on human dendritic cells. Toxicol. In Vitro.

[77] Yang X., Zhang X., Yao Q., Song M., Han Y., Shao B., Li Y. (2019). T-2 toxin impairs male fertility by disrupting hypothalamic-pituitary-testis axis and declining testicular function in mice. Chemosphere.

[78] Tassis P.D., Tsakmakidis I.A., Nagl V., Reisinger N., Tzika E., Gruber-Dorninger C., Michos I., Mittas N., Basioura A., Schatzmayr D. (2020). Individual and combined in vitro effects of deoxynivalenol and zearalenone on boar semen. Toxins.

[79] Agrawal M., Yadav P., Lomash V., Bhaskar A.S.B., Lakshmana Rao P.V. (2012). T-2 toxin induced skin inflammation and cutaneous injury in mice. Toxicology.

[80] Hemmati A.A., Kalantari H., Jalali A., Rezai S., Zadeh H.H. (2012). Healing effect of quince seed mucilage on T-2 toxin-induced dermal toxicity in rabbit. Exp. Toxicol. Pathol..

[81] Cho U.M., Choi J.H., Hwang H.S. (2017). Deoxynivalenol impair skin barrier function through the down regulation of filaggrin and claudin 1/8 in HaCaT keratinocyte. Biotechnol. Bioprocess Eng..

[82] Zhou H., George S., Hay C., Lee J., Qian H., Sun X. (2017). Individual and combined effects of aflatoxin B1, deoxynivalenol and zearalenone on HepG2 and RAW 264.7 cell lines. Food Chem. Toxicol..

[83] Fernández-Blanco C., Elmo L., Waldner T., Ruiz M.-J. (2018). Cytotoxic effects induced by patulin, deoxynivalenol and toxin T2 individually and in combination in hepatic cells (HepG2). Food Chem. Toxicol..

[84] Yu F.-F., Lin X.-L., Wang X., Ping Z.-G., Guo X. (2019). Comparison of apoptosis and autophagy in human chondrocytes Induced by the T-2 and HT-2 Toxins. Toxins.

[85] Yang L., Tu D., Zhao Z., Cui J. (2017). Cytotoxicity and apoptosis induced by mixed mycotoxins (T-2 and HT-2 toxin) on primary hepatocytes of broilers in vitro. Toxicon.

[86] International Agency for Research on Cancer (1993). Monographs on the Evaluation of Carcinogenic Risks to Humans: Some Naturally Occuring Substances: Food Items and Costituents, Heterocyclic Aromatic Amines and Mycotoxins.

[87] European Food Safety Authority (2011). Scientific opinion on the risks for animal and public health related to the presence of T-2 and HT-2 toxin in food and feed. EFSA J..

[88] Poór M., Kunsági-Máté S., Sali N., Kőszegi T., Szente L., Peles-Lemli B. (2015). Interactions of zearalenone with native and chemically modified cyclodextrins and their potential utilization. J. Photochem. Photobiol. B.

[89] Kotowicz N.K., Frac M., Lipiec J. (2014). The importance of *Fusarium* fungi in wheat cultivation-pathogenicity and mycotoxins production: A review. J. Anim. Plant Sci..

[90] Rai A., Das M., Tripathi A. (2020). Occurrence and toxicity of a *fusarium* mycotoxin, zearalenone. Crit. Rev. Food Sci. Nutr..

[91] Zinedine A., Soriano J.M., Moltó J.C., Mañes J. (2007). Review on the toxicity, occurrence, metabolism, detoxification, regulations and intake of zearalenone: An oestrogenic mycotoxin. Food Chem. Toxicol..

[92] Krejcárková A., Šimoník O., Šašková M., Krejčířová R., Drábek O., Rajmon R. (2017). Effects of zearalenone, α-zearalenol, and genistein on boar sperm motility in vitro. Czech J. Anim. Sci..

[93] Harčárová M., Čonková E., Proškovcová M., Falis M. (2020). In vivo assessment of zearalenone toxicity. Folia Vet..

[94] Aichinger G., Pantazi F., Marko D. (2020). Combinatory estrogenic effects of bisphenol A in mixtures with alternariol and zearalenone in human endometrial cells. Toxicol. Lett..

[95] Althali N.J., Hassan A.M., Abdel-Wahhab M.A. (2019). Effect of grape seed extract on maternal toxicity and in utero development in mice treated with zearalenone. Environ. Sci. Pollut. Res..

[96] Yao X., Jiang H., Gao Q., Li Y.-H., Xu Y.N., Kim N.-H. (2020). Melatonin alleviates defects induced by zearalenone during porcine embryo development. Theriogenology.

[97] Cao H., Zhi Y., Xu H., Fang H., Jia X. (2019). Zearalenone causes embryotoxicity and induces oxidative stress and apoptosis in differentiated human embryonic stem cells. Toxicol. In Vitro.

[98] Salem I.B., Boussabbeh M., Neffati F., Najjar M., Abid-Essefi S., Bacha H. (2016). Zearalenone-induced changes in biochemical parameters, oxidative stress and apoptosis in cardiac tissue: Protective role of crocin. Hum. Exp. Toxicol..

[99] Jia Z., Liu M., Qu Z., Zhang Y., Yin S., Shan A. (2014). Toxic effects of zearalenone on oxidative stress, inflammatory cytokines, biochemical and pathological changes induced by this toxin in the kidney of pregnant rats. Environ. Toxicol. Pharmacol..

[100] Szabó A., Szabó-Fodor J., Fébel H., Mézes M., Balogh K., Bázár G., Kocsó D., Ali O., Kovács M. (2018). Individual and combined effects of fumonisin B1, deoxynivalenol and zearalenone on the hepatic and renal membrane lipid integrity of rats. Toxins.

[101] Islam M.R., Kim J.W., Roh Y.-S., Kim J.-H., Han K.M., Kwon H.-J., Lim C.W., Kim B. (2017). Evaluation of immunomodulatory effects of zearalenone in mice. J. Immunotoxicol..

[102] Hueza I.M., Raspantini P.C.F., Raspantini L.E.R., Latorre A.O., Górniak S.L. (2014). Zearalenone, an estrogenic mycotoxin, is an immunotoxic compound. Toxins.

[103] Pistol G.C., Braicu C., Motiu M., Gras M.A., Marin D.E., Stancu M., Calin L., Israel-Roming F., Berindan-Neagoe I., Taranu I. (2015). Zearalenone mycotoxin affects immune mediators, MAPK signalling molecules, nuclear receptors and genome-wide gene expression in pig spleen. PLoS ONE.

[104] Bouaziz C., Sharaf el dein O., El Golli E., Abid-Essefi S., Brenner C., Lemaire C., Bacha H. (2008). Different apoptotic pathways induced by zearalenone, T-2 toxin and ochratoxin A in human hepatoma cells. Toxicology.

[105] Marin D.E., Pistol G.C., Bulgaru C.V., Taranu I. (2019). Cytotoxic and inflammatory effects of individual and combined exposure of HepG2 cells to zearalenone and its metabolites. Naunyn. Schmiedebergs Arch. Pharmacol..

[106] Frizzell C., Ndossi D., Verhaegen S., Dahl E., Eriksen G., Sørlie M., Ropstad E., Muller M., Elliott C.T., Connolly L. (2011). Endocrine disrupting effects of zearalenone, alpha- and beta-zearalenol at the level of nuclear receptor binding and steroidogenesis. Toxicol. Lett..

[107] Wang X., Yu H., Fang H., Zhao Y., Jin Y., Shen J., Zhou C., Zhou Y., Fu Y., Wang J. (2019). Transcriptional profiling of zearalenone-induced inhibition of IPEC-J2 cell proliferation. Toxicon.

[108] Ren Z.H., Deng H.D., Deng Y.T., Deng J.L., Zuo Z.C., Yu S.M., Shen L.H., Cui H.M., Xu Z.W., Hu Y.C. (2016). Effect of the *Fusarium* toxins, zearalenone and deoxynivalenol, on the mouse brain. Environ. Toxicol. Pharmacol..

[109] Jia R., Liu W., Zhao L., Cao L., Shen Z. (2020). Low doses of individual and combined deoxynivalenol and zearalenone in naturally moldy diets impair intestinal functions via inducing inflammation and disrupting epithelial barrier in the intestine of piglets. Toxicol. Lett..

[110] Zhang W., Zhang S., Wang J., Shan A., Xu L. (2020). Changes in intestinal barrier functions and gut microbiota in rats exposed to zearalenone. Ecotoxicol. Environ. Saf..

[111] Lahjouji T., Bertaccini A., Neves M., Puel S., Oswald I.P., Soler L. (2020). Acute exposure to zearalenone disturbs intestinal homeostasis by modulating the Wnt/β-Catenin signaling pathway. Toxins.

[112] Jajić I., Dudaš T., Krstović S., Krska R., Sulyok M., Bagi F., Savić Z., Guljaš D., Stankov A. (2019). Emerging *Fusarium* mycotoxins fusaproliferin, beauvericin, enniatins, and moniliformin in Serbian maize. Toxins.

[113] Tonshin A.A., Teplova V.V., Andersson M.A., Salkinoja-Salonen M.S. (2010). The *Fusarium* mycotoxins enniatins and beauvericin cause mitochondrial dysfunction by affecting the mitochondrial volume regulation, oxidative phosphorylation and ion homeostasis. Toxicology.

[114] European Food Safety Authority (2014). Scientific opinion on the risks to human and animal health related to the presence of beauvericin and enniatins in food and feed. EFSA J..

[115] Jestoi M. (2008). Emerging *Fusarium* -mycotoxins fusaproliferin, beauvericin, enniatins, and moniliformin—A review. Crit. Rev. Food Sci. Nutr..

[116] Devreese M., Broekaert N., De Mil T., Fraeyman S., De Backer P., Croubels S. (2014). Pilot toxicokinetic study and absolute oral bioavailability of the *Fusarium* mycotoxin enniatin B1 in pigs. Food Chem. Toxicol..

[117] Prosperini A., Juan-García A., Font G., Ruiz M.J. (2013). Beauvericin-induced cytotoxicity via ROS production and mitochondrial damage in Caco-2 cells. Toxicol. Lett..

[118] Prosperini A., Font G., Ruiz M.J. (2014). Interaction effects of *Fusarium* enniatins (A, A1, B and B1) combinations on in vitro cytotoxicity of Caco-2 cells. Toxicol. In Vitro.

[119] Olleik H., Nicoletti C., Lafond M., Courvoisier-Dezord E., Xue P., Hijazi A., Baydoun E., Perrier J., Maresca M. (2019). Comparative structure–activity analysis of the antimicrobial activity, cytotoxicity, and mechanism of action of the fungal cyclohexadepsipeptides enniatins and eeauvericin. Toxins.

[120] Agahi F., Font G., Juan C., Juan-García A. (2020). Individual and combined effect of zearalenone derivates and beauvericin mycotoxins on SH-SY5Y Cells. Toxins.

[121] Mamur S., Yuzbasioglu D., Yılmaz S., Erikel E., Unal F. (2018). Assessment of cytotoxic and genotoxic effects of enniatin—A in vitro. Food Addit. Contam. Part A.

[122] Huang C.-H., Wang F.-T., Chan W.-H. (2019). Enniatin B1 exerts embryotoxic effects on mouse blastocysts and induces oxidative stress and immunotoxicity during embryo development. Environ. Toxicol..

[123] Büchter C., Koch K., Freyer M., Baier S., Saier C., Honnen S., Wätjen W. (2020). The mycotoxin beauvericin impairs development, fertility and life span in the nematode *Caenorhabditis elegans* accompanied by increased germ cell apoptosis and lipofuscin accumulation. Toxicol. Lett..

[124] European Commission (2006). Commission Regulation (EC) No. 1881/2006 of 19 December 2006 setting maximum levels for certain contaminants in foodstuffs. Off. J. Eur. Union.

[125] European Commission (2010). European Union Commission Regulation (EU) No. 105/2010 of 5 February 2010 amending Regulation (EC) No 1881/2006 setting maximum levels for certain contaminants in foodstuffs as regards ochratoxin A. Off. J. Eur. Union.

[126] JECFA FAO/WHO (2011). Evaluation of Certain Contaminants in Food Seventy-Second Report of the Joint FAO/WHO Expert Committee on Food Additives.

[127] European Food Safety Authority (2017). Risks to human and animal health related to the presence of deoxynivalenol and its acetylated and modified forms in food and feed. EFSA J..

[128] JECFA FAO/WHO (2002). Evaluation of Certain Mycotoxins in Food. Fifty-Sixth Report of the Joint FAO/WHO Expert Committee on Food Additives.

[129] European Food Safety Authority (2017). Human and animal dietary exposure to T-2 and HT-2 toxin. EFSA J..

[130] JECFA FAO/WHO (2000). Evaluation of Certain food Additives and Contaminants. Fifty-Third Report of the Joint FAO/WHO Expert Committee on Food Additives.

[131] European Food Safety Authority (2016). Appropriateness to set a group health-based guidance value for zearalenone and its modified forms. EFSA J..

[132] Krogh P., Axelsen N.H., Elling F., Gyrd-Hansen N., Hald B., Hyldgaard-Jensen J., Larsen A.E., Madsen A., Mortensen H.P., Moller T. (1974). Experimental porcine nephropathy. Changes of renal function and structure induced by ochratoxin A- contaminated feed. Acta Pathol. Microbiol. Scand. Suppl..

[133] European Food Safety Authority (2020). Risk assessment of ochratoxin A in food. EFSA J..

[134] European Food Safety Authority (2020). Risk assessment of aflatoxins in food. EFSA J..

[135] EU (1999). SCF Opinion of the Scientific Committee on Food on Fusarium-Toxins Part 1: Deoxynivalenol (DON).

[136] European Food Safety Authority (2011). Scientific opinion on the risks for public health related to the presence of zearalenone in food. EFSA J..

[137] Ostry V., Skarkova J., Ruprich J. (2009). Alternaria Mycotoxins in Foodstuffs–Current Information for Health Risk Assessment.

